# The Indian Experience With Nonacog Beta Pegol for the Treatment of Hemophilia B in Children and Adolescents: A Safety and Efficacy Evaluation

**DOI:** 10.7759/cureus.83158

**Published:** 2025-04-28

**Authors:** Nehal Patel, Ghosha Pandav, Rashmi Thanvi, Priyanka Solanki, Dipti Shah, Anil Katara

**Affiliations:** 1 Department of Pediatrics, Gujarat Medical Education and Research Society (GMERS) Medical College Hospital, Sola, Ahmedabad, IND

**Keywords:** abr, hemophilia b, nonacog beta pegol, prophylaxis, target joints

## Abstract

Purpose: Regular prophylactic factor replacement is recommended as optimal therapy for hemophilia B. Factor concentrate with longer half-lives would allow successful prophylaxis as less frequent dosing would be less burdensome for patients and caregivers. Nonacog Beta Pegol (N9-GP), a glycopegylated factor IX with an extended half-life, has been established globally. However, real-world data from India remain limited. This study evaluates the safety, efficacy, and feasibility of low-dose N9-GP prophylaxis in Indian children/adolescents with hemophilia B.

Methods: In this retrospective study, a total of seven patients with hemophilia B were included. Baseline assessments of the annualized bleeding rate (ABR), Functional Independence Score in Hemophilia (FISH), and Hemophilia Joint Health Score (HJHS) were conducted for all patients. Dosing was individualized (mean 34.76 IU/kg; range: 25-40 IU/kg every three weeks) based on patient weight, baseline ABR, and joint status, in accordance with resource-conscious protocols suitable for Indian settings. Patients were monitored for any adverse events throughout the study period. After 12 months of treatment, the ABR, FISH, and HJHS were reassessed and compared with baseline values. Screening for inhibitors was performed for all patients at the end of the 12-month period.

Results: The mean age was 11.2 years, ranging from four years to 18 years. The mean overall ABR before using N9-GP was 21.1 (range 12 - 48) bleeds/year. After treatment with N9-GP, the mean overall ABR was 0.42 (0-1) bleeds/year. Six patients had target joints before starting treatment. At the end of 12 months of prophylaxis, only one patient had a target joint involving one joint with mobility and swelling significantly improved in the affected joint. Four patients remained bleed free on N9-GP prophylaxis. Three patients experienced minor bleeding once, which was just before the next dose of prophylaxis. Baseline mean HJHS was 26.71/124 (range 10 - 47). After 12 months on N9-GP, mean HJHS was 7.5/124 (range 0-16). Before treatment, the mean FISH score was 23.8/32 (range 17-29). After treatment, the mean FISH score was 31.4/32 (range 28 - 31). None of them developed any adverse drug reactions or thromboembolic events during the entire course of treatment. None of the patients developed inhibitors.

Conclusion: N9-GP has been shown to be well tolerated, safe, and efficacious in the treatment and prevention of bleeding episodes in people with hemophilia B.

## Introduction

Hemophilia B is a rare genetic disorder, with an estimated incidence of 1 in 30,000 male births. Like hemophilia A, hemophilia B also leads to spontaneous bleeding, particularly in joints, muscles, and internal organs, resulting in pain, disability, and reduced quality of life [1]. Despite advances in treatment, hemophilia management remains a significant challenge, especially in developing countries like India, where access to healthcare limits the treatment options [2].

N9-GP, a recombinant factor IX with glycopegylated modification that prolongs its half-life, is a valuable treatment option for hemophilia B patients. This extended half-life factor can increase the half-life by fivefold compared to the standard factor IX. It has been extensively studied through the PARADIGM clinical trial program, which investigated the safety and efficacy of N9-GP across diverse patient populations for prophylaxis, on-demand treatment, and in perioperative settings, a comprehensive development pathway evaluating its safety, efficacy, and long-term outcomes across diverse patient populations. The trials have demonstrated that N9-GP is effective in preventing bleeding episodes, minimizing joint damage, and significantly improving quality of life for individuals with hemophilia B. Because of the longer half-life of N9-GP, it also offers the benefit of achieving the clinical outcomes, with reduced frequency of injections compared to standard half-life factors [3-8].

According to Annual Global Survey 2020, only 9% and 4% of children and adults with hemophilia were on prophylaxis, respectively [9]. Studies have pointed out a higher degree of disability in patients with hemophilia in India. India is one of the countries with a large number of patients diagnosed with hemophilia in the world, and the majority of these patients are on episodic treatment rather than the standard of care prophylaxis because of resource constraints, leading to increased morbidity and mortality [10].

Against this backdrop, this case series aims to highlight the efficacy and safety of low-dose N9-GP prophylaxis in children and adolescents with hemophilia B. By investigating the use of low-dose N9-GP, we hope to provide a more accessible and affordable treatment option for patients in India and similar resource-constrained settings. Our study will assess the impact of low-dose N9-GP on bleeding frequency, joint health (HJHS), and functional ability (FISH), providing valuable insights into the potential of this treatment approach.

## Materials and methods

Study design

This retrospective observational study conducted between 13th August 2021 (first patient first visit) and 23rd May 2023 (last patients last visit) involves seven children and adolescents with severe hemophilia B, who were receiving prophylaxis with standard half-life (SHL) Factor IX, who were switched to extended half-life factor (N9-GP) at baseline, at a tertiary care center in Gujarat. Patient data were retrieved from medical records (MR).

Study population

The study included patients diagnosed with hemophilia B who were aged 18 years or younger and had initiated prophylaxis with N9-GP; written informed consent was obtained from adult participants or from the legal guardians of minor participants. Patients with a history of inhibitors, incomplete medical records, or those who did not provide informed consent were excluded. 

Baseline data included the age and weight of the patients, as well as their treatment history. The dose and frequency of injections were documented, with patients receiving N9-GP at a dose of 34.76 IU/kg (range: 25-40 IU/kg) every three weeks for one year. Joint status and bleed status were assessed at baseline, with annualized bleed rates calculated from the number of bleeds in the preceding year. Joint status was evaluated using the Hemophilia Joint Health Score (HJHS) version 2.1, and functional independence was assessed with the Functional Independence Score in Hemophilia (FISH) score, both at baseline and at 12 months after initiation of N9-GP. After 12 months of treatment, data regarding the overall bleeds, spontaneous bleeds, and traumatic bleeds were recorded and their relation to treatment administration was noted. Data regarding target joint resolution was collected at the end of the observation period by the HJHS score and the FISH score.

Scoring and interpretation

Annualized Bleeding Rate (ABR)

The ABR is defined as follows:

Total bleeds during the observation period / Duration (in years). A lower ABR indicates better bleeding control.

HJHS 2.1 Score

The HJHS 2.1 score (range: 0 to 124) assesses joint health, including swelling, muscle atrophy, range of motion, and pain. Higher scores indicate worse joint damage. It includes a total score, joint-specific scores, and a global gait score.

FISH Score

The FISH score (range: 0 to 32) evaluates functional independence in daily activities. Higher scores reflect better physical function. Each activity is graded from 1 to 4 based on the amount of assistance required, and the score is determined through direct observation rather than self-reported ability.

There is no categorization (e.g., mild, moderate, severe) for either the HJHS or FISH scores.

The results are presented in descriptive form with mean and standard deviation. Inhibitor status of the patients was assessed throughout the observation period based on the routine monitoring plan. 

## Results

Demographics

Patients who were receiving prophylaxis with standard half-life (SHL) Factor IX and who were switched to extended half-life factor (N9-GP) at baseline are shown in Table 1. The mean age of the study patients was 11.2 years, with the youngest four years and the eldest 18 years (Table 2).

**Table 1:** Dose and frequency of SHL prophylaxis. EHL: Extended Half-Life; SHL: Standard Half-Life

Patients	(SHL) Before prophylaxis IU/KG/year	(EHL) After prophylaxis IU/KG/year
Patient 1	960	561
Patient 2	960	637
Patient 3	1200	765
Patient 4	960	629
Patient 5	960	425
Patient 6	480	680
Patient 7	400	425

**Table 2:** Baseline characteristics of patients.

Baseline Features	Patient 1	Patient 2	Patient 3	Patient 4	Patient 5	Patient 6	Patient 7
Age (Years)	18	17	14	13	9	5	4
Weight (Kg)	45	61	23	42	40	25	20
Frequency of dosing (weekly)	3	3	3	3	3	3	3
Dose (IU/Kg)	33.33	37.5	45	37.5	25	40	25
Bleeding sites	Knee joint	Knee joint	Joint bleed, hematuria	Ankle joint	Muscle bleeds	Joint	Muscle, joint
Target joints at baseline	One	One	Three	One	One	Two	Zero

ABR outcomes

**Figure 1:**
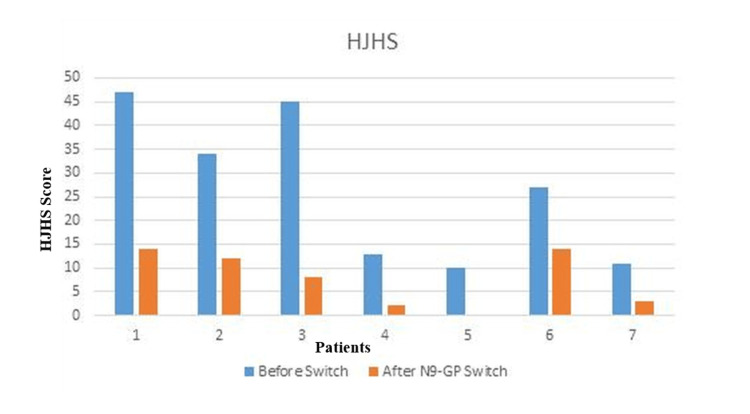
HJHS score before and after switching to N9-GP. HJHS: Hemophilia Joint Health Score; N9-GP: Nonacog Beta Pegol

**Figure 2:**
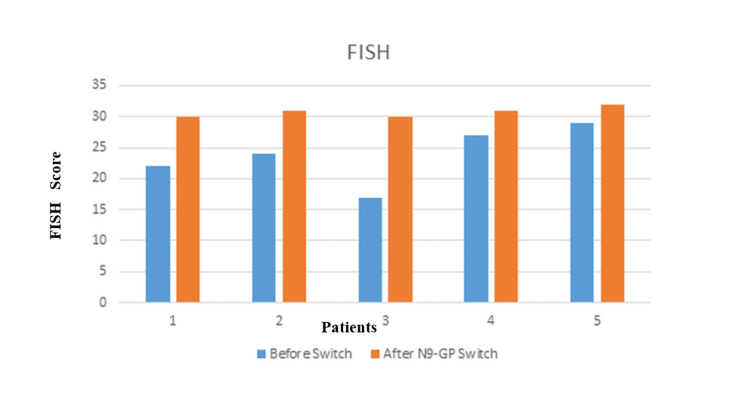
FISH score before and after switching to N9-GP. FISH: Functional Independence Score in Hemophilia; N9-GP: Nonacog Beta Pegol

The mean overall ABR before using N9-GP was 21.1 (12 - 48) bleeds/year. N9-GP reduced the mean ABR from 21.1 to 0.42 bleeds/year, with 4/7 patients achieving zero bleeds. Three patients experienced minor bleeds pre-dose, suggesting potential for further dose optimization. Six patients had target joints before starting treatment. At the end of 12 months of prophylaxis, only one patient had target joint involving one joint with mobility and swelling improved (Figures 1, 2).

Four patients remained bleed free on N9-GP prophylaxis. Three patients presented with minor bleeds once before the next dose of prophylaxis. The bleeds were resolved with the standard treatment protocol.

Joint health & function

The baseline mean HJHS was 26.71/124 (10 - 47) which improved to mean of 7.5/124 (0-16) after 12 months on N9-GP. Before treatment, the mean FISH score was 23.8/32 (17-29) which also improved to mean FISH of 31.4/32 (28 - 31) after treatment (Table 3).

**Table 3:** Bleeding profile of patients after switching to N9-GP. Before is “before switching to EHL therapy”, After is “After switching to EHL”, Baseline is the day of “switching to EHL” EHL - Extended Half-Life; N9-GP: Nonacog Beta Pegol; ABR: Annualized Bleeding Rate

		Patient 1	Patient 2	Patient 3	Patient 4	Patient 5	Patient 6	Patient 7
Overall ABR	Before	24	24	30	24	24	12	10
	After	0	1	0	1	0	1	0
Spontaneous ABR	Before	24	24	30	24	24	12	10
	After	0	1	0	1	0	0	0
Traumatic ABR	Before	0	0	0	0	0	0	0
	After	0	0	0	0	0	1	0
Bleed resolution after 1 or 2 injections. (Y/N)		-	Y	-	Y	-	Y	-
Target joints after N9GP Prophylaxis		0	0	1	0	0	0	0
Remaining Bleed free after N9 GP Prophylaxis (Y/N)		Y	N	Y	N	Y	N	Y

Safety profile

None of them developed any adverse drug reactions or thromboembolic events. None of the patients developed inhibitors. The dose of N9-GP used was 25-45 IU per kg with individualization.

## Discussion

N9-GP 40 IU/kg/week is approved for prophylactic management of patients with hemophilia B [10,11]. However, low-dose prophylaxis is important in the context of resource-limited countries like India. An earlier study in India has shown that low-dose prophylaxis is better than on-demand treatment in children with hemophilia; it has shown not only to reduce the bleeds but also improve schooling and reduce hospital admissions [12]. Further health economic analysis has also shown that in Indian children, intermediate prophylaxis is better compared to on-demand treatment [13]. Studies done in other parts of the world also show that low-dose prophylaxis can provide a cost-effective option and play an important role in improving hemophilia care in developing countries [14,15]. At the end of 12 months of N9-GP prophylaxis, four patients remained bleed free, and three patients experienced minor bleeds once, just before the next dose of prophylaxis. Limited data on the efficacy of lower doses of 24N9 GP, especially in Indian patients, and the positive results from this study highlight the need for exploring dose individualization in patients with hemophilia B.

There are many studies that highlight the benefits of switching from standard half-life factors to extended half-life factors. Switching to extended half-life factors has been shown to reduce ABRs and reduce injection frequency and overall factor consumption [16,17]. An earlier real-world evidence study from Canada has shown that switching to N9-GP, irrespective of the previous product SHL or other EHLs, has been shown to reduce the median ABR and median factor consumption [18]. The Hemophilia Joint Health Score (HJHS) was specifically developed by the Physical Therapy Expert Working Group of the International Prophylaxis Study Group to detect early joint changes in boys aged 4 to 18 years with hemophilia. The current HJHS version 2.1 comprises an assessment of specific features, or items, of the six index joints and an assessment of global gait. For each of the six joints, the following items are scored: swelling (scored 0‐3), duration of swelling (0‐1), muscle atrophy (0‐2), crepitus on motion (0‐2), flexion loss (0‐3), extension loss (0‐3), joint pain (0‐2), and strength (0‐4). The maximum score for an individual index joint is 20. Gait is scored 0 to 4. The maximum HJHS total score is 124, with a higher score indicating worse joint health [19]. The HJHS reduced from 26.71/124 (range 10-47) to 7.5/124 (range 0 - 16) in the seven study participants after 12 months of low-dose N9 GP prophylaxis. Similarly, we have seen an improvement in the FISH score in patients who switched from SHL to N9-GP in our study. This is one of the first studies to report the improvement in HJHS after N9-GP prophylaxis in India. Similarly, we have seen an improvement in the FISH score in patients who switched from SHL to N9-GP in our study. HJHS improvements (26.71 → 7.5) and target joint resolution in 6/7 patients suggest N9-GP’s joint-protective effects, though the small sample warrants cautious interpretation. Even in this patient, the joint mobility and swelling were improved compared to the baseline.

At the end of 12 months of N9 GP prophylaxis, four patients remained bleed free, and three patients experienced minor bleeds once, just before the next dose of prophylaxis. Our study has shown resolution in the target joint in all but one patient. Even in this patient, the joint mobility and swelling were improved compared to the baseline.

Limitations

Though this descriptive case series has highlighted several advantages of using an individualized plan for managing patients with hemophilia, it has a small sample size, and without a control group, it is hard to apply the findings broadly. We propose doing a study involving multiple centers across India, probably register-based, involving a large number of patients.

## Conclusions

Low-dose N9-GP prophylaxis significantly reduced bleeding and improved joint health in this Indian cohort, offering a feasible option for resource-limited settings. Larger studies are needed to validate dosing strategies. N9-GP has reduced bleeding episodes with less frequent injections, thus reducing infusion burden and facilitating adherence to prophylaxis.
